# Dorsal Dislocation of the First and Second Metatarsophalangeal Joint: A Case Report and Literature Review

**DOI:** 10.7759/cureus.45407

**Published:** 2023-09-17

**Authors:** Georges F Bassil, Fadi Nader, Achraf Lajmi, Zied Missaoui

**Affiliations:** 1 Orthopedic Surgery, Grand Hôpital de l'Est Francilien - Site de Meaux, Meaux, FRA

**Keywords:** plantar, dorsal, first ray, hallux, sesamoid bone, dislocation, joint, mtp

## Abstract

Dorsal dislocation of the toes is an infrequent injury that can result in severe pain and deformity. Timely diagnosis and appropriate management are paramount for optimizing patient outcomes. This case report illustrates a 53-year-old male patient who suffered dorsal dislocation of the first and second metatarsophalangeal (MTP) joints due to a crush injury. We present the clinical manifestation, radiographic findings, and management approach for this unique isolated first and second ray MTP joint dorsal dislocation, without any associated fractures. This case report underscores several critical observations: firstly, hallux dorsal dislocation can potentially coincide with other injuries; secondly, it can stem from crushing trauma to the big toe; and thirdly, successful closed reduction, when followed by effective immobilization and early rehabilitation, can yield outstanding outcomes. Additionally, the report emphasizes the importance of pursuing another closed reduction attempt under general anesthesia, if the initial attempt in the emergency room proves unsuccessful, before contemplating open reduction.

## Introduction

Dorsal dislocation of the first and second metatarsophalangeal (MTP) joints combined is an infrequent yet clinically significant injury that primarily arises from traumatic incidents, such as crush injuries or direct impacts to the foot [1]. In addition to its occurrence in sports and accidental events, it is crucial to acknowledge the relevance of this injury in occupational settings. Occupations that involve physical labor, such as construction workers or individuals working in warehouses or factories with heavy machinery and equipment, face an increased risk of sustaining this injury and should be always equipped with appropriate protective footwear. First MTP dislocation can tremendously affect the patient's ability to bear weight and walk properly. Jahss describes two cases in 25,000 patients (incidence of 0.008%) and Giannikas et al. report four cases in 10,000 patients (incidence of 0.04%) [1,2]. The rarity of this injury has resulted in limited literature on its diagnosis and management strategies. It is worth noting that certain case reports have demonstrated positive results, even in cases of first MTP joint dislocations that were neglected and reduced several months after the initial injury [3]. Timely diagnosis remains paramount to achieving optimal patient outcomes, as well as minimizing potential complications such as osteopenia, residual osteoarthritis, and avascular necrosis of the metatarsal head. To facilitate the understanding and management of these unique injuries, Jahss categorized these dislocations into two fundamental types [1]. Type I is a dorsal dislocation of the MTP joint and sesamoid bone with rupture of the capsule; however, the intersesamoid ligament and the sesamoid bone remain intact. Type II dorsal dislocations are either associated with a disruption of the intersesamoid ligament (Type IIA) or involve a transverse fracture of either sesamoids (Type IIB). There is also a Type IIC classification that was proposed by Copeland et al., which represents a combination of both complete disruption of the intersesamoid ligament and a transverse fracture of either sesamoid [4]. However, this classification does not cover all types of first MTP joint dislocation nor is reliable in predicting the feasibility of closed reduction [5]. In the subsequent sections, we will delve into the detailed case presentation, classification systems, therapeutic interventions, and post-treatment rehabilitation strategies. By sharing our experience and discussing the relevant literature, we hope to enhance the understanding of this injury's intricacies and contribute to informed clinical decision-making.

## Case presentation

**Figure 1:**
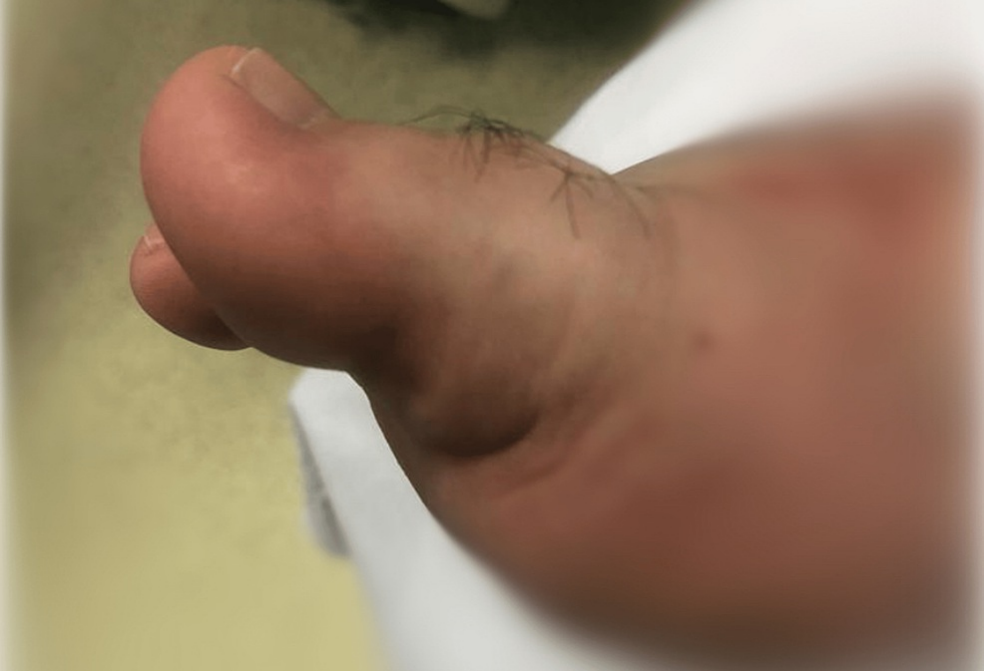
Clinical image showing the dorsal dislocation of the hallux at first presentation at the emergency department.

A 53-year-old male patient presented to the emergency department with severe pain, swelling, and deformity in his right first and second toes after being crushed by a pallet truck. Clinical pictures of the dislocated big toe are shown in Figure 1.

**Figure 2:**
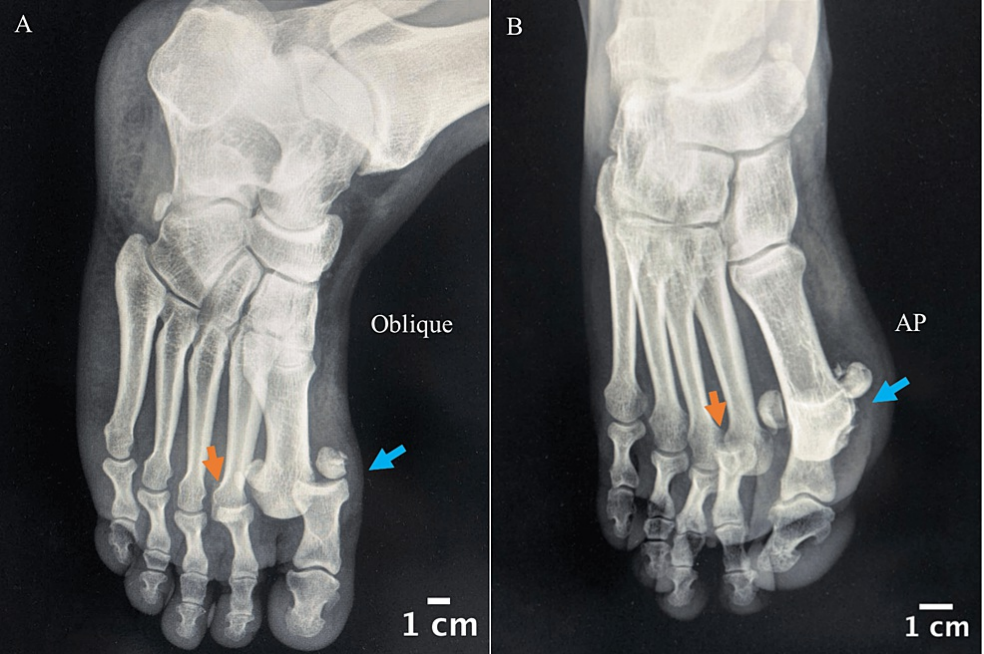
Oblique and AP view of the patient's right foot showing dorsal dislocation of the first (blue arrows) and second (red arrows) toes MTP joints. AP: Anteroposterior; MTP: Metatarsophalangeal.

An initial radiograph (Figure 2) in the emergency department revealed complete dorsal dislocation of both the first and second MTP joints. Closed reduction of the second toe was successfully performed in the emergency department under midazolam and morphine. However, the reduction of the first toe's dorsal dislocation was unsuccessful, necessitating transfer to the operating room for further intervention.

**Figure 3:**
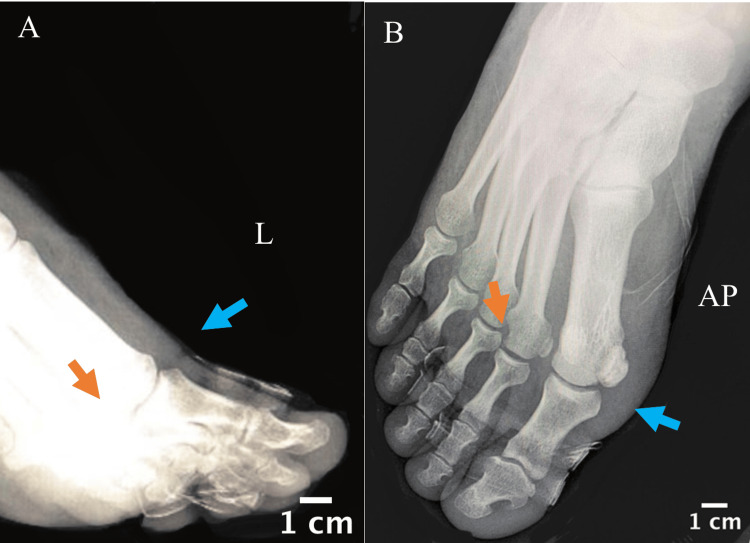
X-rays lateral (A) and anteroposterior (B) views after closed reduction of the first toe dorsal dislocation under general anesthesia followed by buddy taping; arrows point to the reduced first (blue arrow) and second (red arrows) MTP joints. MTP: Metatarsophalangeal.

In the operating room, the patient underwent a reduction of the first toe MTP under general anesthesia. Fluoroscopic guidance was utilized to ensure a satisfactory reduction of the joint and the sesamoid. The reduction was successful, and joint stability was confirmed. Postoperative X-rays were done in the operating room to confirm satisfactory reduction (Figure 3). Buddy taping of the first, second, and third toes was done to maintain and protect reduction, and the patient was provided with appropriate pain management.

**Figure 4:**
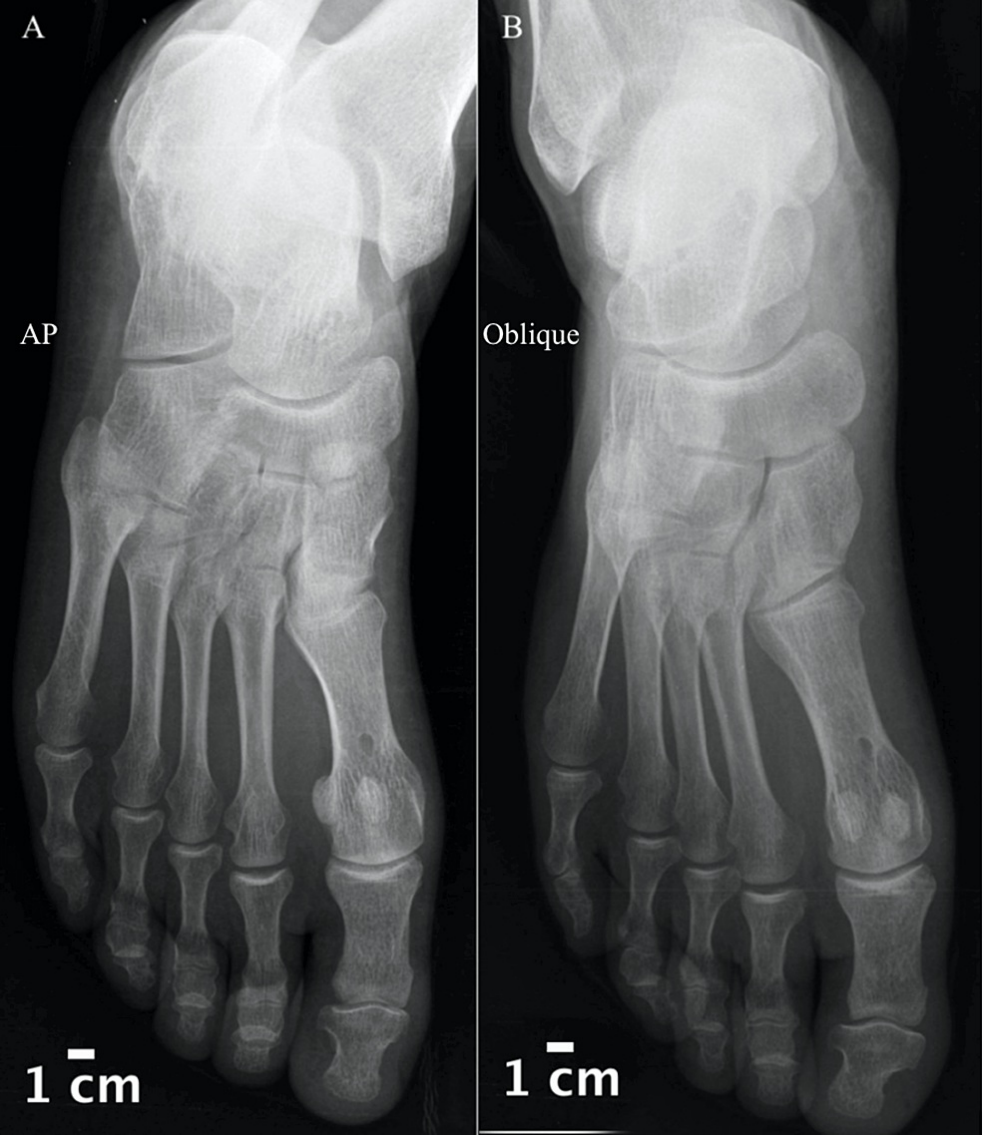
X-rays of anteroposterior (A) and oblique (B) views at one year of follow-up.

At one-month follow-up patient was full weight bearing with slight limping and had pain at the end of the arc of motion when doing passive or active dorsiflexion/plantar flexion. There were no varus or valgus instability, the patient was started on passive, active assisted, and active range of motion physiotherapy sessions three times per week. At the two-month follow-up patient was able to walk without limping and to do full active and passive range of motion of the big toe and was comfortable wearing shoes with a wide toe box but still reported some episodes of mild pain at the end of his working day or when doing exertional activity. At the six-month follow-up, the patient was able to resume his regular daily activity wearing regular footwear with minimal to no pain. Follow-up X-rays at one year are shown in Figure 4. At the two-year follow-up, the patient was able to run without pain; however, he still reported some episodes of mild pain in his big toe at the end of the evening after a long working day.

## Discussion

**Figure 5:**
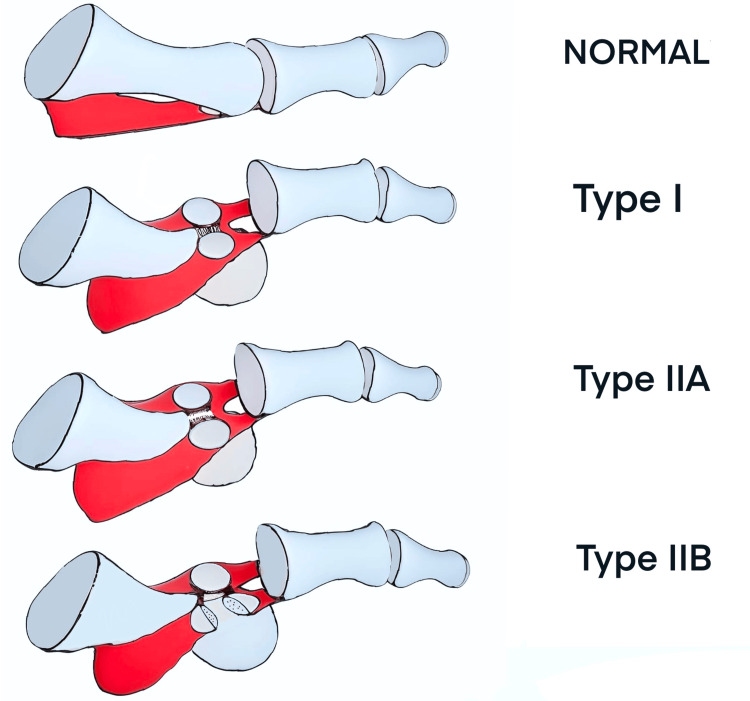
Jahss classification system of first MTP joint dorsal dislocation. MTP: Metatarsophalangeal.

Dorsal dislocation of the MTP joint is a rare but potentially debilitating injury that requires prompt and appropriate management. This unique injury occurs due to a forceful hyperextension of the MTP joint of the first toe, often resulting from incidents such as motor vehicle accidents or falls from a height [6,7]. In our case, the dislocation occurred following a crushing injury to the first and second toes by a pallet truck, slightly differing from the classical mechanism of forceful hyperflexion described by Jahss [1]. To effectively categorize and manage dorsal dislocations of the toes, the Jahss classification system (Figure 5) among other classification systems is commonly employed.

This classification system divides the injuries into two main types (Type I and Type II) based on the direction of displacement and the involvement of the sesamoid bones [1]. Type I dislocations occur when an axial load is applied to the plantar aspect of the distal end of the first ray while the first MTP joint is in a hyper-dorsiflexed position. The sesamoid bones remain intact, but the dislocation happens as the capsule ruptures, leading to the dorsal displacement of the phalanx and sesamoids. This type is anatomically like the complex dislocation of the second metacarpal joint described by Kaplan [8]. On the other hand, Type II dislocations result from further dorsiflexion force, which can cause either the intersesamoid ligament to rupture (Type IIA) or a transverse fracture of one of the sesamoids, usually the medial one (Type IIB). There is also a Type IIC classification that was proposed by Copeland et al. [4], which represents a combination of both complete disruption of the intersesamoid ligament and a transverse fracture of either sesamoid. In our case the patient's dorsal dislocation of the MTP joint of the hallux best fit in the Type IIA. The injury involved complete rupture of the intersesamoid ligament, but without a transverse fracture of the sesamoid bone. According to Jahss, Type I dislocations often require surgical intervention if closed reduction fails [1]. However, Type II dislocations respond well to closed reduction under local anesthesia or sedation. The surgical reduction is guided by careful assessment of the sesamoid position on plain radiography, which helps determine the appropriate treatment plan. In our case, closed reduction in the emergency department was attempted but without success. As a result, the management plan involved closed reduction under general anesthesia followed by buddy taping of the first three toes together.

The recent review of various reported traumatic dislocations of the first MTP joint has highlighted a limitation in the Jahss classification system, as it currently does not adequately predict the feasibility of closed reduction for these injuries and does not cover all the types of first ray MTP joint dislocation. To address this and encompass all the different types of big toe MTP joint dislocations (dorsolateral, lateral, medial, and plantar dislocation) and their associated treatment approaches, a more comprehensive classification system was proposed by Zrig et al. in 2017 [5]. This updated classification system divides the first ray MTP joint dislocations into three types. Type I, Dorsal Dislocation of the First MTP Joint includes three subtypes. In Type IA, the intersesamoid ligament is intact and the sesamoidal complex is not dislocated. Closed reduction is possible and typically yields favorable outcomes with appropriate management. In Type IB, the intersesamoid ligament remains intact but the sesamoidal complex is dislocated over the metatarsal neck. Closed reduction is not feasible in this case and open reduction is necessary to achieve proper realignment. Type IC involves a true discontinuity of the intersesamoid ligament either due to tearing or avulsion. Surprisingly, closed reduction is still possible in Type I dislocation which may offer a relatively favorable prognosis. Type II dorsal dislocation includes Lateral and Medial Dislocations of the First MTP Joint and has two subtypes. Type IIA, pure lateral dislocation. Closed reduction in most cases is possible and can lead to successful joint realignment. Type IIB, dorsolateral and dorsomedial dislocations. Closed reduction remains a viable option for managing Type II dislocations effectively. Type III, Plantar Dislocation of the First MTP Joint. Surprisingly, closed reduction is possible in Type III, which offers a promising treatment option for achieving proper joint alignment. Based on this classification system our patient's first MTP dislocation best fit into Type IC.

The reduction of a dorsal dislocation of the MTP joint can be achieved using both closed and open techniques. For closed reduction, a common method involves applying axial traction and dorsally directed pressure over the plantar aspect of the head of the proximal phalanx while the foot is stabilized [5]. This maneuver aims to overcome the hyperextension force that caused the dislocation and realign the joint into its proper position. However, Garcia Mata et al. noted that direct pressure on the phalanx with hyperextension should be applied without axial traction to prevent any incarceration [9]. Successful closed reduction leads to immediate relief of the clinical deformity and restoration of the joint's alignment. In our case, the reduction in the emergency department under benzodiazepines was not successful most probably because the patients’ muscles were not relaxed enough and the reduction had to be performed under general anesthesia. In cases where closed reduction proves to be unsuccessful, surgical intervention becomes necessary to achieve anatomical reduction and joint stability. Various surgical approaches have been described in the literature. One technique by Younis et al. involves an open reduction with intact collateral ligaments: using a dorsal approach to the dislocated toe the surgeon will apply axial traction and use a Blunt Hohmann retractor from under the intersesamoid ligament on the head of the first metatarsal to lever the proximal phalanx back into its place on the head of the first metatarsal [10]. After reduction, the toes are tested and if unstable, K-wire fixation is needed. Another open reduction technique utilizes a plantar approach, which allows for successful reduction of the first MTP joint as well as better visualization and repair of the plantar plate without the need for a K-wire fixation; however, it can induce weight-bearing pain and trophic disorders [11]. Plantar dislocation of the hallux is a different but related injury that can occur at the first MTP joint. The treatment approach for plantar dislocation of the hallux is similar to dorsal dislocations and may involve closed reduction or surgical intervention, depending on the severity of the dislocation and the stability of the joint. It is important also to mention a unique and effective reduction technique for plantar dislocation of the hallux described by Bohl et al. [12]. The authors performed an intra-articular injection of sterile saline into the MTP joint through a 20-gauge needle dorsally. Surprisingly, this simple procedure resulted in near-immediate and spontaneous resolution of the clinical deformity without the need for additional reduction force.

Both closed and open reduction procedures for dislocated toes carry certain risks of complications. After closed reduction, potential complications include incomplete reduction, soft tissue injury, fractures, joint stiffness, and recurrent dislocation. Open reduction may be associated with infection, delayed healing, neurovascular injury, orthopedic hardware complications, wound healing issues, joint stiffness, and adhesions. Close postoperative monitoring and adherence to rehabilitation protocols can help minimize the risk of complications and optimize recovery outcomes. The prognosis of dislocation of the first MTP joint depends on the dislocation type and the quality of management. Although a good outcome is often seen, some complications, such as osteoarthritis, hallux rigidus, osteonecrosis of the metatarsal head, neuroalgodystrophy, and sesamoid pseudarthrosis, have been reported [5]. In the case of sesamoid pseudarthrosis, sesamoid excision should be used only in the presence of significant functional discomfort. Early diagnosis, appropriate reduction techniques, and starting the rehabilitation process as soon as the joint is stable can significantly impact the success of the management and reduce the risk of future complications in these challenging injuries. It is worth mentioning cases of dorsal dislocation of the MTP joint of the hallux that share similarities with our case report (Table 1).

**Table 1:** Cases of dorsal dislocation of the first MTP joint that share similarities with our case report. MTP: Metatarsophalangeal; N/A: Cannot be classified using the Jahss classification system.

Authors	Case presentation	Jahss type	Treatment
Al-Mohrej et al. 2017 [3]	Neglected dorsolateral dislocation of the first metatarsophalangeal joint in a 46-year-old man.	Type III	Open reduction using dorsal approach and fixation with K-wires.
Mizumoto et al. 2021 [13]	Dislocation of the first metatarsophalangeal joint concomitant with Lisfranc joint dislocation in a 45-year-old man due to a severe pinch point crush injury.	N/A	Repair of the torn Lisfranc and tarsometatarsal ligament and open reduction of the first MTP joint using medial approach.
Sharma et al. [14]	First and Second Metatarsophalangeal Joint Open Dislocations in a 19-year-old due to road traffic accident.	Type II	Open reduction of the first and second MTP joint utilizing the existing plantar wound.
Hussain et al. 1999 [15]	Dislocation of the First Metatarsophalangeal Joint with Fracture of Fibular Sesamoid in a 36-year-old man who struck his toe against the door sill.	Type I	Reduction of the first MTP joint through dorsal approach.
Younis et al. 2017 [10]	First MTP joint dislocation treated with open reduction without fixation in a 33-year-old female patient involved in a motor vehicle accident.	Type I	Open MTP joint reduction using dorsal approach without K wire pinning.
Bohl et al. 2018 [12]	Incarcerated Plantar Dislocation of the First Metatarsophalangeal Joint in a 26-year-old male sustained a hyperplantarflexion force to his first left MTP joint while attempting to “crack” his great toe.	N/A	Reduction of the first MTP joint plantar dislocation using intra-articular saline injection.
Zrig et al. 2017 [5]	Dislocation of the First Metatarsophalangeal Joint in a 32-year-old, healthy, male biker that fell from his motorcycle.	N/A	Closed reduction of the MTP joint under nitrous oxide analgesia.

## Conclusions

In conclusion, Jahss Type IIA dorsal dislocation of the first MTP concomitant with dorsal dislocation of the second toe due to a crushing injury, while rare, presents a significant clinical challenge. While Jahss and other classification systems have provided valuable insights into the categorization of these injuries, a more comprehensive classification system proposed by Makram Zrig and co-authors offers a promising approach to encompassing the various types of dislocations and predicting the feasibility of closed reduction. In our case, the patient was treated by closed reduction under general anesthesia for the first toe, while the second toe was successfully reduced in the emergency department. Postoperative follow-up and early rehabilitation led to a favorable outcome, with the patient regaining full function and minimal to no pain while doing his daily activities. The management of these injuries can involve closed or open reduction techniques, depending on the type and severity of the dislocation. Complications can arise from both closed and open reduction procedures, using the appropriate classification system will help in choosing the appropriate treatment. This case report enhances our understanding of managing this uncommon injury by emphasizing a few crucial observations. It highlights the potential co-occurrence of hallux dorsal dislocation with other injuries, and the fact that MTP dorsal dislocation can result from a crushing trauma to the big toe, and demonstrates that successful closed reduction, when followed by effective immobilization and early rehabilitation, can result in satisfactory outcome in these types of cases.
